# Preparing for the COVID-19 Pandemic From a Community Hospital Perspective: Team of Teams Approach

**DOI:** 10.7759/cureus.11387

**Published:** 2020-11-08

**Authors:** Matthew Mo Kin Kwok, Eliza Chan, Joseph Copeland, Eric Juneau, Andrew Smith

**Affiliations:** 1 Emergency Medicine, Faculty of Medicine, University of British Columbia, Vancouver, CAN; 2 Emergency Medicine, Richmond Hospital, Vancouver Coastal Health, Richmond, CAN

**Keywords:** emergency medicine, covid-19, pandemic, emergency preparedness

## Abstract

This report describes one community hospital emergency department’s (ED's) experience in preparing for the COVID-19 pandemic. In order to mitigate the impact of the pandemic on both our community and our ED, several proposals were reviewed. Strategies were employed to ensure the protection of ED staff and to lessen the impact of potential patient volume surges. A plan was agreed upon using the “team of teams” approach. Using this method, we achieved our goal of having a plan in place to manage the impact of the pandemic and safely care for our patients.

## Introduction

Our hospital is a university-affiliated community teaching hospital located in Richmond, British Columbia, Canada. We receive approximately 60,000 emergency department (ED) visits per year and we are the closest hospital to the Vancouver International Airport which sees over 25 million passengers from over 100 countries annually [1]. As a result, we frequently encounter returning travelers or visitors from other countries.

On December 31, 2019, the Wuhan Municipal Health Commission, China, reported a cluster of cases of pneumonia in Wuhan, Hubei Province. A novel strain of coronavirus was identified and later named SARS-CoV-2, the cause of the disease now called COVID-19. On January 14, 2020, a case of COVID-19 was confirmed in Thailand, the first recorded case outside China [2]. On January 25, 2020, a man who arrived in Toronto, Ontario from Wuhan was the first documented case of COVID-19 in Canada [3]. On March 11, 2020, the World Health Organization classified the global outbreak of COVID-19 as a pandemic [2].

Disaster medicine can be divided into three broad categories: mass-casualty incidents, hazardous-material accidents, and more rarely, pandemics. All three categories are low-frequency, high-stake events that require collaborative, well-coordinated responses. By definition, disasters are situations that overwhelm the day-to-day operations of a given setting. A system can often step up to address challenges that are above day-to-day expectations. The inability to address the extra challenge is the issue in disaster. Whether it is the number, acuity, or spacing of patients, disasters stress normal operations. Successfully managing these challenges requires both preparation and practice.

## Materials and methods

**Figure 1:**
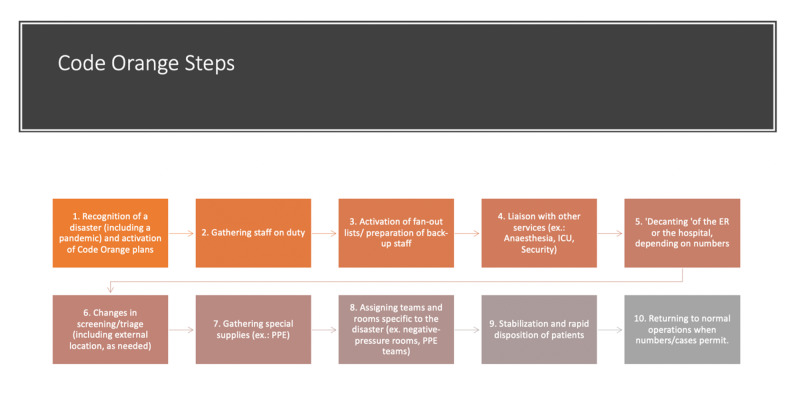
Code Orange steps

In British Columbia, accredited hospitals usually have a Code Orange disaster plan. General principles of disaster planning are straightforward (Figure 1), and generic plans can be found online [4]. Code Orange plans should be tailored to the needs and skills of each specific setting and should be updated from time to time. In order for the plan to be effective, input from frontline workers is critical.

In the early months of 2020, the impact of COVID-19 on our community and our ED was unknown. Knowledge of the various presentations, complications, and treatments for COVID-19 continued to evolve. In addition to the regular review of current literature, as a group of emergency physicians serving the community, we began to plan for responses to increases in volume and acuity driven by COVID-19. We believed it was our responsibility to have strategic plans for the potential stresses on our local health delivery system. As a result of the categorization of issues and analysis of our system of care, we agreed on the plan to build teams to effectively work collaboratively. In the results section, we highlight three teams that were formed in our ED in preparation for the COVID-19 pandemic.

## Results

“Team of teams”

CODE 66/Simulation Team

One of the main concerns voiced by the group was ensuring the safety management of COVID-19 patients, particularly during endotracheal intubation, while maintaining excellent clinical care. Initial reports on COVID-19 emphasized the severe respiratory failure of some patients and the requirement to mechanically ventilate approximately 5% of admitted patients [5]. Furthermore, intubations are among the highest risk procedures with regards to staff exposure to respiratory infection [6]. All patients requiring intubation during the pandemic were assumed to be COVID-19 positive.

**Figure 2:**
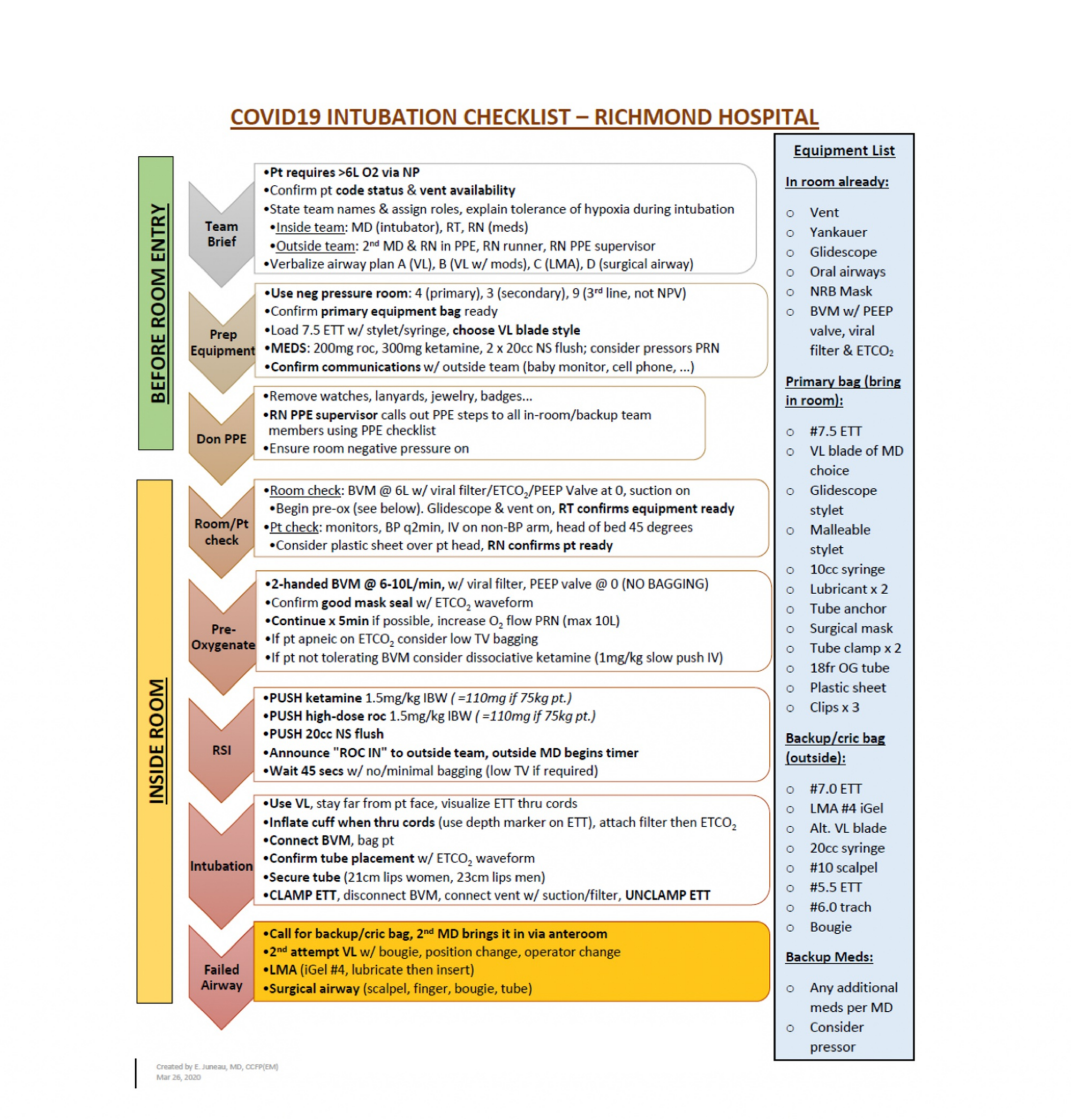
COVID-19 endotracheal intubation checklist

To address these concerns, a team of four emergency physicians was assembled to create a local intubation protocol based on the latest available evidence (Figure 2). The team came up with the protocol for intubation then led, in conjunction with nursing educators, a series of intubation simulations in our ED. Throughout this process, 42 physicians from emergency medicine, anesthesia, and intensive care participated in simulations, along with the majority of the ED nursing staff and respiratory therapists. The first simulation took place 12 days after COVID-19 was declared a pandemic.

Concurrently, this team of emergency physicians liaised with the anesthesiologists at our hospital to develop a dedicated team of physicians for all emergency intubations and resuscitations of potential COVID-19 patients. This led to the development of a “Code 66” team to respond to any patient in the hospital developing respiratory failure. This team also assumed responsibility for all cardiac arrest (code blue) responses within the hospital. The members of this team managed multiple critically ill patients and performed daily simulations of intubation and cardiac arrest scenarios with ED and ward staff.

The priority of the “Code 66” team was to implement safe and evidence-based protocols for the initial management of the most critically ill COVID-19 patients. We prioritized the use of simulations to achieve proficiency in employing the protocol. The process was well-received by frontline staff and administration because it had full engagement by all levels of our hospital organization.

Reserve Physicians Team

The reserve physicians team was tasked with mitigating potential physician shortages to cover the needs of the ED. We had existing procedures in place for calling the current roster of 39 emergency physicians in the event of a critical surge in patient volumes or physician illness. However, we believed that a contingency plan was needed to recruit other physicians to be placed on a reserve list. This was done in anticipation of increased patient volumes due to COVID-19, as well as an emergency physician shortage due to illness or quarantine.

A team of three emergency physicians worked together to address this issue. We began with the following questions:

(i) What constitutes an ideal physician candidate who would be part of the reserve physicians group?

(ii) What would the scope of practice of the reserve physicians be in our ED?

(iii) When should we trigger and deploy the reserve physicians?

(iv) How would we fund this group of physicians?

We decided to assemble a group of ten family physicians with admitting privileges at our hospital who were available and willing to work in our ED. They would manage patients with stable vital signs and non-critical issues. Patient volume and wait time triggers for calling upon the reserve physicians were formalized. Funding would come from contingent COVID-19 funds from the health authority.

Within two weeks we were able to recruit ten family physicians who met our criteria. The reserve physicians were oriented to our department and ready to be deployed within three weeks. A system was set up to call upon the reserve physicians if needed.

Virtual Health Team

Addressing surges in patient volume and building system capacity with limited and fixed clinical resources have been a longstanding challenge contributing to overcrowding in many EDs [7]. Pressure on already strained resources including beds, equipment, and other supplies as well as personnel will be accentuated during a pandemic. Maintaining a motivated clinical team capable of complex decision-making over prolonged periods, while balancing unclear but finite risks to self and family is critical to maintaining a cohesive department. The COVID-19 pandemic has the potential to reduce the availability of clinical personnel for a variety of reasons such as shortages of personal protective equipment (PPE), quarantine, etc. Acute care telemedicine operating on the principle of “good-enough” represents one alternative that could enable both urban and rural EDs to complement and maintain critical staffing levels despite the ongoing pandemic.

**Figure 3:**
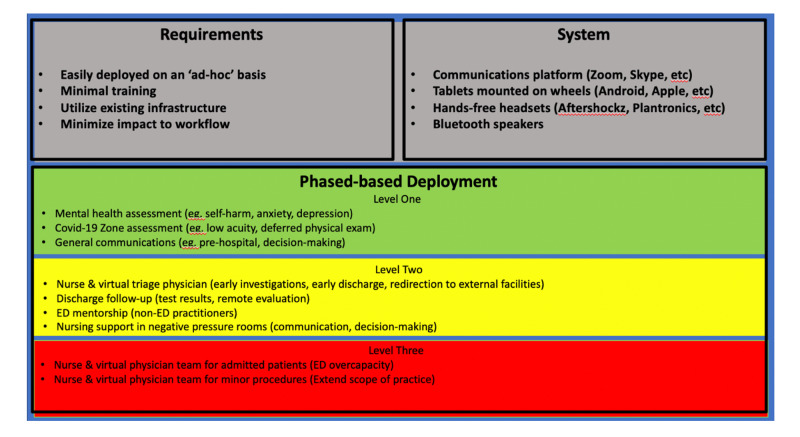
Virtual health proposed requirements, concept system, and phased deployment

**Figure 4:**
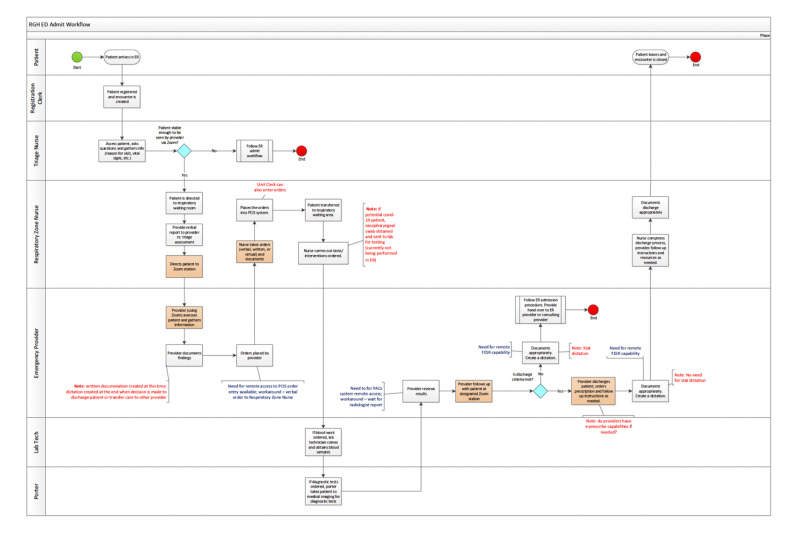
Process map of patient flow

As a part of our ED’s multifaceted pandemic planning approach, we explored low-cost telemedicine as an option to augment staffing while addressing ED surge capacity. Proposed requirements, concept systems, and phased deployment were established (Figure 3). A sample patient flow was developed by a team of analysts at our health authority (Figure 4).

A virtual health approach has the benefit of reducing PPE utilization and reducing the risk of clinician exposure to COVID-19 by limiting direct patient encounters in low-risk cases, which is a controversial, yet pragmatic approach given the limited utility of physical examination in select settings. A potential challenge of implementing virtual care in the ED pertains to physician compensation. Each health authority would need to address the question of funding requirements and reimbursement particularly given a myriad of compensation models.

## Discussion

**Figure 5:**
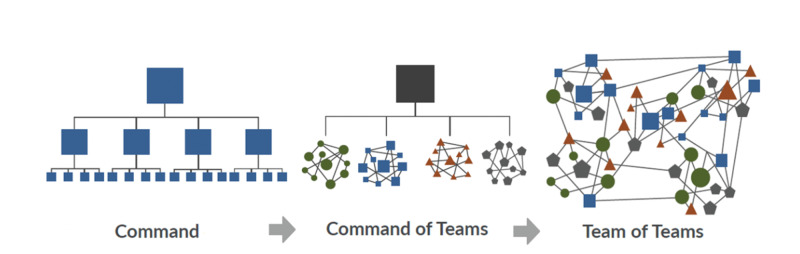
"Team of teams" approach

“Team of teams” is a concept developed by Stanley McChrystal [8], a retired United States Army General who took command of the Joint Special Operations Task Force in Iraq in 2003. He discovered that despite having an army with a massive advantage in numbers, equipment, and training, the conventional “top-down” approach of military tactics was failing due to its deficiency of speed and flexibility. As a result, McChrystal et al. developed what has been coined the “team of teams ” approach [8]. Instead of waiting for organization leaders to make decisions, frontline team members set up teams formulating solutions to problems that the organization faces (Figure 5). These teams would collaboratively work together towards a common goal, thus fully addressing the issues encountered by the organization. 

In our situation, a group of emergency physicians recognized the unpredictability and rapidity of change occurring in healthcare delivery due to the COVID-19 pandemic. We focused on the organizational aim of providing exceptional and timely care to our patients while keeping our staff safe during the pandemic. As a result, we took it upon ourselves to strategically deploy frontline line staff in a “team of teams” model to break down the issues, analyze our system, and build solutions.

## Conclusions

The “team of teams” approach is one way of managing healthcare planning during the COVID-19 pandemic. In this approach, we were able to develop our strategy using a “ground-up” method, which was then communicated to other teams within the hospital, and subsequently to other hospitals within our regional health authority. Capitalizing on the interconnectedness of these webs of teams, this approach allowed us to respond rapidly to the changing landscape of uncertainties and complexities presented by the pandemic.

The key to our success was our team’s ability to communicate openly and effectively. Building trust, accepting vulnerabilities, and celebrating strengths were all paramount in building a strong organization that can adapt, respond, and react in a timely manner. As our world becomes more integrated and complex, we will have to devise ways of building effective and resilient teams to face future healthcare challenges. Our ED has demonstrated one model of how this can be accomplished.
